# Onset-to-treatment time and aneurysmal regression predict improvement of cranial neuropathy after flow diversion treatment in patients with symptomatic internal carotid artery aneurysms

**DOI:** 10.1136/jnis-2022-019202

**Published:** 2022-07-19

**Authors:** Ryo Akiyama, Akira Ishii, Takayuki Kikuchi, Masakazu Okawa, Yukihiro Yamao, Yu Abekura, Isao Ono, Natsuhi Sasaki, Hirofumi Tsuji, Hirotoshi Imamura, Taketo Hatano, Nobuyuki Sakai, Susumu Miyamoto

**Affiliations:** 1 Department of Neurosurgery, Kyoto University Graduate School of Medicine Faculty of Medicine, Kyoto, Kyoto, Japan; 2 Department of Neurosurgery, Kobe City Medical Center General Hospital, Kobe, Hyogo, Japan; 3 Department of Neurosurgery, Kokura Memorial Hospital, Kitakyushu, Fukuoka, Japan

**Keywords:** Aneurysm, Flow Diverter, Cranial nerve

## Abstract

**Background:**

Although flow diversion plays a pivotal role in treating internal carotid artery aneurysms presenting with cranial neuropathy, predictors of symptom improvement have not been established.

**Objective:**

To investigate improvement of symptoms after flow diversion treatment in patients with internal carotid artery aneurysms causing cranial neuropathy, with sufficient follow-up period. Additionally, to examine factors associated with improvement of symptoms.

**Methods:**

This retrospective multicenter study examined patients with unruptured internal carotid artery aneurysms presenting with cranial neuropathy who were treated using flow diversion and followed up for at least 12 months. Study outcomes were transient worsening of symptoms and symptom status 12 months after treatment. Patient and aneurysm characteristics were statistically analyzed.

**Results:**

Seventy-seven patients were included. Data needed for outcome analysis were available for 66 patients. At the 1-, 3-, 6-, 12-month, and last follow-ups, the proportion of patients with resolved or improved symptoms was 26% (20/77), 51% (39/77), 74% (57/77), 83% (64/77), and 79%(62/77), respectively. Symptom onset-to-treatment time <6 months (OR=24.2; 95% CI 3.09 to 188.84; p=0.002) and aneurysmal regression (OR=23.1; 95% CI 1.97 to 271.75; p=0.012) were significantly associated with symptom improvement. Transient symptom worsening and worse symptoms at 12 months occurred in 19/77 (25%) and 2/77 (3%) patients, respectively.

**Conclusions:**

The rate of cranial neuropathy symptom improvement after flow diversion increased over the first 12 months after treatment, but not thereafter. Treatment within 6 months of symptom onset and aneurysmal regression were predictors of symptom improvement.

**What is already known on this topic**

- Flow diversion plays a pivotal role in treating internal carotid artery aneurysms presenting with cranial neuropathy, but predictors of symptom improvement in these patients have not been established.

**What this study adds**

- Cranial neuropathy symptoms after flow diversion treatment improved over time in the first 12 months, but not thereafter; treatment within 6 months of symptom onset and aneurysmal regression were associated with improvement.

**How this study might affect research, practice or policy**

- This study provides a more accurate reflection of symptom improvement after flow diversion treatment of internal carotid artery aneurysms causing cranial neuropathy and suggests ways to improve cranial neuropathy outcome in real-world clinical practice.

## Introduction

Large and giant cavernous and paraclinoid internal carotid artery aneurysms often cause cranial neuropathy that affect cranial nerves (CNs) II, III, IV, V, and VI.^1–3^ Conventional treatments, such as surgical clipping and endovascular coiling, have advantages and disadvantages. Although clipping can immediately achieve complete aneurysmal regression, the complication rate is high.^4^ Coiling has a lower complication rate but is associated with worse neuro-ophthalmological outcomes owing to mass effect from the coil mass as well as incomplete occlusion and a higher recanalization rate.^5 6^

A new approach to treatment of large and giant cavernous and paraclinoid aneurysms has recently emerged—flow diversion, which can achieve a high occlusion rate without requiring adjunctive coil placement while minimizing complications.^7^ Flow diversion is conceptually an ideal treatment for symptomatic aneurysms, as it can achieve complete aneurysmal regression without placing a metallic implant within the aneurysmal sac. In clinical practice, flow diversion has been playing a pivotal role in treating symptomatic aneurysms.^8–10^ However, rate of symptom improvement and related factors vary between reports.^11–14^ Although cranial neuropathy symptoms after flow diversion appear to improve over time,^11^ follow-up periods in previous studies have not been adequate.^11–14^ This may explain the inconsistency between reports. Moreover, symptoms may be evaluated by different examiners before and after treatment. Predictors of symptomatic course have yet to be identified.^13^

This study aimed to investigate symptom improvement after flow diversion treatment in patients with internal carotid artery aneurysms causing cranial neuropathy with sufficient follow-up period. We also aimed to examine factors associated with improvement of symptoms. To ensure consistent evaluation of cranial neuropathy, neurological assessment was performed by three examiners using a standardized symptom scale before and after treatment.

## Methods

### Ethics approval

This retrospective multicenter study was approved by the institutional review boards of Kyoto University Hospital (ID R0058; Multicenter Neuroendovascular Therapy Registry), Kobe City Medical Center General Hospital, and Kokura Memorial Hospital. Informed consent was obtained in the form of an opt-out on the institutional website.

### Data collection

All patients who undergo flow diversion treatment of unruptured aneurysms in the participating institutions are registered in a prospectively maintained database. Three Japanese cerebrovascular centers contributed to the use of patient data. Data for the period from June 2015 to December 2020 were retrospectively reviewed. Patients who presented with cranial neuropathy (CNs II, III, IV, V, and VI) because of an internal carotid artery aneurysm, were treated with flow diversion, and were followed up for at least 12 months were eligible for inclusion. Clinical data were obtained from the database and patient medical records.

### Treatment strategy and endovascular procedure

Patients received 100 mg/day aspirin and 75 mg/day clopidogrel for 10 to 14 days before the procedure. Platelet function testing was routinely performed using the VerifyNow P2Y12 assay and the VerifyNow Aspirin assay (Accumetrics, San Diego, California, USA) on the day before. Antiplatelet medications were adjusted accordingly, as in previous reports.^15^ Dual antiplatelet therapy was continued for at least 6 months after the procedure, and single antiplatelet therapy was continued indefinitely thereafter.

All procedures were performed under general anesthesia using the standard transfemoral approach. Heparin anticoagulation was implemented throughout the procedure. Flow diversion treatment was performed using standard technique, as described previously.^15 16^

Flow diverter (FD) implantation was performed by three neuroendovascular specialists (AI, HI, NoS), each of whom has more than 10 years of experience in intracranial stent placement. The selection of FD type and number was at the discretion of the operator.

In principle, adjunctive coil embolization was performed if the aneurysm was located in the subarachnoid space to prevent delayed rupture. The volume embolization rate (VER), the ratio of the volume of the packed coils to the aneurysm volume, was calculated in patients who underwent adjunctive coiling.

### Angiographic follow-up

Angiographic outcome was assessed with digital subtraction angiography or magnetic resonance angiography 6 and 12 months after the procedure. Other MRI sequences were also performed: fluid-attenuated inversion recovery, T2*, diffusion-weighted imaging, and T1 black blood (T1 BB). Imaging studies were assessed by a blinded radiologist and neurosurgeon. Aneurysm occlusion was categorized according to the O’Kelly–Marotta (OKM) grading scale.^17^ Aneurysm size was assessed 12 months after the procedure using T1 BB MRI. If T1 BB was not available, another sequence was used. Aneurysm volume was calculated as π × (D1×D2×D3)/6, with D1, D2, and D3 representing the largest aneurysm diameter in the axial, sagittal, and coronal planes, respectively. If the variation rate was >30%, variation of aneurysm size was identified.

### Clinical assessment and follow-up

Cranial neuropathy was assessed 1, 3, 6, and 12 months after the procedure. Thereafter, follow-up was continued every 6 to 12 months at the discretion of the operator. Neurological assessment was performed by three examiners using the same symptom scale before and after treatment to assure consistency. Subjective symptoms were also recorded. Whenever possible, an additional neuro-ophthalmologic evaluation was also performed during follow-up visits. Symptoms after treatment were described relative to those before treatment as resolved, improved, stable, or worse. Transient worsening of symptoms was defined as a return to, or improvement in, baseline symptoms at 12 months after initial worsening.

Steroid use and dosage were at the operator’s discretion. In general, prednisolone was typically initiated at a dose of 1 mg/kg and then tapered off according to response in patients who experienced worsening of symptoms. Steroid use was defined as the use of prednisolone ≥10 mg over the 12-month follow-up period.

### Outcomes

Study outcomes were cranial neuropathy status 12 months after treatment compared with baseline, and incidence of transient worsening.

### Statistical analyses

Statistical analyses were performed using JMP Pro software version 16 (SAS Institute, Cary, North Carolina, USA). Continuous data are presented as medians with IQR and were compared using the Wilcoxon rank sum test. Categorical data are presented as numbers with percentage and were compared using the Fisher exact test. Variables found to be significantly associated with improvement in cranial neuropathy in univariate analysis were further evaluated using multivariable logistic regression. A p value <0.05 was considered significant.

## Results

### Patient and aneurysm characteristics

Seventy-seven patients met the inclusion criteria for the study (77 aneurysms). Data regarding comorbidities, cranial nerves affected, time from symptom onset to treatment, and changes in aneurysm size on MRI were not available for 11 patients. Therefore, background information necessary for outcome analysis could be obtained for 66 patients (66 aneurysms). Patient and aneurysm characteristics are summarized in table 1. Median age was 69 years (range 20–88). Fifty-seven patients (86%) were women and 9 (14%) were men. Comorbidities included hypertension in 37 patients (56%), dyslipidemia in 25 (38%), diabetes in 4 (6%), and a history of smoking with Brinkman index >100 in 21 (32%). Aneurysm location was cavernous internal carotid artery in 50 patients (76%), paraclinoid internal carotid artery in 15 (23%), and internal carotid artery–posterior communicating artery in 1 (2%). Median maximum aneurysm diameter was 18.7 mm (range 7.7–34.8). Median neck size was 8.1 mm (range 1.4–15.4). Twenty-eight aneurysms (42%) had a maximum diameter greater than 20 mm. The total number of cranial neuropathies was 89. Neurological symptoms were related to CNs II, III, IV, V, and VI in 22, 27, 1, 11, and 28 patients, respectively; 16 patients (24%) had symptoms related to more than one CN. Median time from symptom onset to treatment was 4 months (range 0–81). Among the 44 patients who presented with oculomotor dysfunction, six (14%) had complete paralysis and 38 (86%) had partial paralysis. Mean number of FDs implanted per patient was 1.2 (range 1–5); multiple FDs were implanted in 10 patients (15%). The FDs implanted were the Pipeline embolization device (PED; Medtronic Neurovascular, Irvin, California, USA) in 76 patients (99%) and the Flow Redirection Endoluminal Device (FRED; Microvention, Aliso Viejo, California, USA) in one (1%). Adjunctive coiling was performed in 18 patients (27%): two had a cavernous segment aneurysm and 16 had a paraclinoid segment or internal carotid artery–posterior communicating artery aneurysm. Median VER in the 18 patients who underwent coiling was 14.1% (range 6.1%–26.7%).

**Table 1:** Patient and aneurysm characteristics

Variable	n=66
Age (years)	69 (57–75)
Women	57 (86)
Comorbidities	
Hypertension	37 (56)
Dyslipidemia	25 (38)
Diabetes mellitus	4 (6)
History of smoking	21 (32)
Aneurysm characteristics	
Aneurysm size (mm)	18.7 (16.0–23.0)
Aneurysm neck (mm)	8.1 (6.1–10.7)
Aneurysm location	
Cavernous portion	50 (76)
Paraclinoid portion	15 (23)
ICA-PC	1 (2)
Cranial neuropathy (n=89)	
CN II	22 (25)
CN III	27 (30)
CN IV	1 (1)
CN V	11 (12)
CN VI	28 (31)
Multiple cranial neuropathy	16 (24)
Symptom onset-to-treatment (months.)	4 (2–9)
Procedure characteristics	
Multiple stents used	10 (15)
Adjunctive coiling	18 (27)
VER (%)	14.1 (10.7–17.5)
Clinical follow-up (months.)	39 (26–55)
Steroid use	19 (29)
Aneurysm occlusion at 6 months	
OKM grade: D	27 (41)
OKM grade: C-D	49 (74)
Aneurysm occlusion at 12 months.	
OKM grade: D	46 (70)
OKM grade: C-D	56 (85)
Morbidity	
Symptomatic hemorrhagic stroke	1 (2)
Symptomatic ischemic stroke	1 (2)
30-Day major stroke	0 (0)
Re-treatment	8 (12)

Values shown are medians (IQR) or numbers (%).

CN, cranial nerve; ICA-PC, internal carotid artery–posterior communicating artery; OKM, O’Kelly–Marotta; VER, volume embolization rate.

### Angiographic follow-up

The rate of OKM grade D occlusion (no aneurysm filling) at 6 and 12 months was 41% and 70%, respectively. The rate of OKM grade C (small neck remnant) or D occlusion at the same time points was 74% and 85%, respectively.

### Complications and re-treatment

Ipsilateral symptomatic intracerebral hemorrhage occurred as a complication in one patient (2%). Ipsilateral symptomatic embolic ischemic cerebral infarction occurred in another (2%). Major stroke, defined as deterioration of two or more points in the modified Rankin scale, did not occur in any patient in the first 30 days after treatment.

Re-treatment was performed in eight patients (12%) at a median of 16 months (range 9–26) after the initial procedure; all but one were re-treated more than 1 year later. Re-treatment consisted of overlapping the same type of FD as used in the initial treatment; however, in one patient, overlapping did not occlude the aneurysm so parent artery occlusion was performed.

### Clinical follow-up

Median clinical follow-up was 39 months (range 12–72). Neuro-ophthalmologic evaluation was performed prior to treatment in 47 of 66 patients (71%); neuro-ophthalmologic follow-up was available in 29 (44%). Nineteen patients (29%) received steroids after treatment.

**Figure 1:**
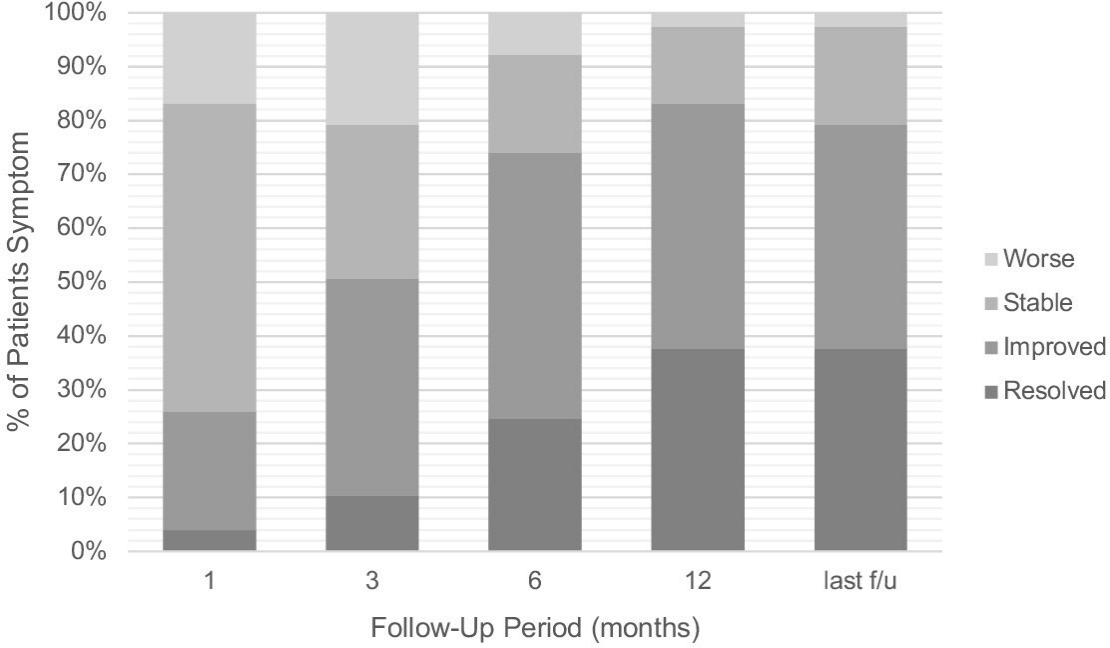
Cranial neuropathy outcome 1, 3, 6, and 12 months after treatment and at last follow-up. Median last follow-up was 39 months (IQR 26–55).

Figure 1 shows the course of cranial nerve symptoms over time after treatment. At the 1-, 3-, 6-, 12-month and last follow-ups, the proportion of patients with resolved or improved symptoms was 26% (20/77), 51% (39/77), 74% (57/77), 83% (64/77), and 79% (61/77), respectively.

Transient worsening occurred in 19 patients. All cases of transient worsening occurred within 3 months of treatment; 11 patients (58%) had worsening at 1 month. Among these 19 patients, 15 (79%) had improved by 6 months and 4 (21%) by 12 months.

The incidence of resolution or improvement of symptoms at 12 months stratified by involved CN was 55% (12/22) for CN II, 85% (23/27) for CN III, 0% (0/1) for CN IV, 100% (10/10) for CN V, and 93% (27/29) for CN VI. When evaluating CNs III, IV, and VI together as a unit (oculomotor nerves), the incidence of symptom resolution or improvement was 88% (50/57). Compared with other cranial neuropathies, the incidence of symptomatic improvement was significantly lower for CN II neuropathies (p=0.001) and significantly higher for CN VI neuropathies (p=0.048; online supplemental table 1).

10.1136/jnis-2022-019202.supp1 Supplementary data

### Outcomes

Resolution or improvement of cranial neuropathy symptoms at 12 months was observed in 64 patients (83%): 29 (38%) had symptom resolution and 35 (45%) had improvement. We were able to obtain the necessary data for outcome analysis in 66 of the 77 patients. Results of univariate and multivariate analyses of predictors of symptom resolution or improvement are summarized in table 2. In the univariate analyses, factors significantly associated with resolution or improvement included were not having diabetes (p=0.034), onset-to-treatment time <6 months (p=0.002), adjunctive coiling (p=0.003), and aneurysmal regression (p=0.0002). Multivariate analysis of these factors showed that onset-to-treatment time <6 months (OR= 24.2; 95% CI 3.09 to 188.84; p=0.002) and aneurysmal regression (OR=23.1; 95% CI 1.97 to 271.75; p=0.012) were independent predictors of cranial neuropathy resolution or improvement.

**Table 2:** Univariate and multivariate analyses for predictors of resolution and improvement of cranial neuropathy

Variable	Univariate			Multivariate		
	Improved	Unimproved	P value	OR	95% CI	P value
No. of patients	51	15				
Age (years)	69 (57–75)	66 (55–75)	0.613			
Women	46 (90)	11 (73)	0.192			
Hypertension	30 (59)	7 (47)	0.555			
Dyslipidemia	19 (37)	6 (40)	1.000			
Diabetes mellitus	1 (2)	3 (20)	0.034	25.8	0.06 to 11 739.28	0.298
Smoker	17 (33)	4 (27)	0.758			
Aneurysm size ≥20 mm	21 (41)	7 (47)	0.771			
Aneurysm neck ≥8 mm	28 (55)	6 (40)	0.384			
Complete paresis	4 (11)	2 (33)	0.182			
Onset-to-treatment <6 months	36 (71)	3 (21)	0.002	24.2	3.09 to 188.84	0.002
Steroid use	16 (31)	3 (20)	0.524			
Adjunctive coiling	9 (18)	9 (60)	0.003	5.9	0.77 to 45.08	0.087
Aneurysm occlusion*						
6 Months	40 (78)	9 (60)	0.185			
12 Months	45 (88)	11 (73)	0.217			
Shrinkage of aneurysm	32 (63)	1 (7)	0.0002	23.1	1.97 to 271.75	0.012

Values shown are medians (IQR) or numbers (%).

*Aneurysm occlusion is defined as O’Kelly–Marotta grade C or D.

Cranial neuropathy was worse 12 months after treatment in two patients (3%). The two patients with worsening at 12 months had aneurysm enlargement and underwent re-treatment. On the other hand, 64 patients without worsening at 12 months had aneurysm enlargement in 1 (2%) and re-treatment in 6 (9%). Nineteen patients (25%) experienced transient worsening. When univariate analysis was performed, factors associated with transient worsening of symptoms were maximum aneurysm diameter ≥20 mm (p=0.010) and steroid usage (p=0.001).

## Discussion

To the best of our knowledge, this is the largest series to investigate outcomes after flow diversion treatment in patients with internal carotid artery aneurysms presenting with cranial neuropathy. In addition, no previous study has reported detailed long-term follow-up of symptoms in these patients in a real-world clinical setting. In our study, as in a previous report,^11^ the proportion of patients whose symptoms resolved or improved increased over the first 12 months. After 12 months, this proportion decreased slightly because of symptom worsening in two patients with a partially occluded aneurysm that had recurred or enlarged. Previous studies have reported that the occlusion rate increases over the first 12 months in aneurysms treated using flow diversion, but not much after that time.^18 19^ Considering all the above, additional treatment may be advisable for patients treated with flow diversion who do not experience improvement in cranial neuropathy and aneurysm occlusion within the first 12 months of treatment. Furthermore, patients with improving symptoms but only partial aneurysm occlusion at 12 months may later experience worsening symptoms because of aneurysm recurrence or enlargement. Early re-treatment may be advisable rather than observation in such cases.

### Improvement of cranial neuropathy after flow diversion treatment

In our study, 79% of patients had resolution or improvement of cranial neuropathy symptoms at last follow-up, which is equivalent to previous studies and reconfirms the efficacy of flow diversion treatment for symptomatic aneurysms. Previous studies reporting cranial neuropathy after flow diversion treatment are summarized in online supplemental table 2)^20 21^ ^22 23^. Across all studies, the overall rate of improvement was 72% (318/444). For each cranial nerve, the rates of improvement for CN II; CNs III, IV, and VI combined; and CN V were 59%, 74%, and 74%, respectively. In our study and previous studies, CN II was found to have worse outcomes than other CNs. This may be because of confounding factors owing to the large number of patients who underwent adjunctive coiling or due to the vulnerability of CN II itself. However, another possibility is that nerve compression might have been present before symptoms were recognized because visual field defects are often difficult to notice; therefore, treatment might have been delayed.

### Factors related to improvement

A previous study reported that cranial neuropathy outcome after flow diversion treatment is better if the time from onset to treatment is under 1 month^11^; however, our study showed that symptoms can improve with an even longer onset-to-treatment time (<6 months). It is difficult to start treatment less than 1 month after symptom onset owing to the need for preoperative examinations and antiplatelet therapy. The fact that treatment within a 6-month window can still result in symptom improvement is useful to relieve the anxiety of both patients and clinicians.

Improvement of cranial neuropathy after flow diversion treatment was associated with aneurysmal regression. An analysis of factors related to aneurysmal regression is shown in online supplemental table 3). Aneurysm occlusion was associated with aneurysmal regression. Previous reports have shown that aneurysm occlusion is associated with symptom improvement.^12^ Although aneurysmal occlusion was not associated with cranial neuropathy improvement in our study, it may be considered an important factor associated with symptom improvement. In addition, our analysis showed that aneurysmal regression was difficult to obtain with adjunctive coiling. Also, univariate analysis showed that adjunctive coiling was not associated with symptom improvement. Indeed, adjunctive coiling seems to worsen the prognosis of cranial neuropathy symptoms. A previous study found that adjunctive coiling is not associated with cranial neuropathy outcome after flow diversion treatment^11^; however, because VER was not reported, it is possible that loose coil packing might have affected their results. Because the range of VER was wide in our study, we performed additional analysis to investigate whether VER affected the rate of symptom improvement (online supplemental table 4). The improvement rate was low when the VER was high, especially ≥13%. A previous study of symptomatic aneurysms treated with FD and loose coil packing (VER <12%) reported high rates of aneurysmal regression and symptom improvement,^14^ which is consistent with our findings. Aneurysms located in the subarachnoid space are at risk of delayed rupture after flow diversion treatment and adjunctive coiling may prevent this.^24 25^ Because the presence of symptoms due to an aneurysm mass effect is a risk factor for delayed rupture,^24^ symptomatic aneurysms located in the subarachnoid space undergoing flow diversion treatment should also be coiled to prevent delayed rupture. Loose coil packing (target VER <13%) may improve cranial neuropathy outcome when treating such aneurysms.

### Factors related to worsening and transient worsening

Several previous studies have analyzed factors related to cranial neuropathy improvement, but only one has analyzed symptom worsening or transient worsening. Transient symptom worsening seems to be unique to flow diversion treatment and occurs frequently.^8^ In our study, transient worsening occurred in 25% of patients. As in previous reports,^13^ we found that transient worsening was more common in patients with aneurysm size ≥20 mm. Clinicians should be careful when treating large symptomatic aneurysms. We also found that steroid use was associated with transient worsening. However, this was because steroids were used in patients whose symptoms worsened: the association was non-causal. Rather, steroids presumably alleviate symptoms because the steroids lessen the inflammatory response caused by rapid thrombosis of the aneurysm. In patients who experienced transient worsening, MRI often showed rapid thrombosis within the aneurysm and high T2 signal intensity in the tissue adjacent to the aneurysm, suggesting that thrombosis-related inflammation had spread to the CNs. In most of these cases, aneurysmal occlusion was achieved and aneurysmal regression was frequent. In other words, if we can overcome the early inflammatory phase, mass effect should resolve and symptoms should improve. Therefore, we believe that use of steroids for such cases is warranted to improve cranial neuropathy outcome. It should be cautioned that steroids are not effective for cranial neuropathy symptoms related to aneurysmal mass effect.

All the patients with cranial neuropathy worsening at 12 months had aneurysm enlargement and required re-treatment. Several predictors of incomplete aneurysm occlusion after flow diversion treatment have been established (older age, large aneurysm size, branching artery arising from the aneurysmal dome, and fusiform aneurysm).^26 27^ Clipping, trapping with bypass, adjunctive coiling with low packing density, and parent artery occlusion may be useful in symptomatic aneurysms with these characteristics, as incomplete occlusion is the main reason for re-treatment.

### Limitations

This study has several limitations. Although it used prospectively collected data, the analyses were retrospective, which is associated with inherent limitations. The multivariate analysis might have had insufficient power because of the relatively small number of patients in whom symptoms did not improve. Although occlusion rate, degree of paralysis, and age were not examined in the multivariate analysis because they were not significant in the univariate analyses, they have been previously reported as factors associated with symptom prognosis. Therefore, they might have been confounders. Finally, some patients with cranial neuropathy in our study did not have neuro-ophthalmologic follow-up.

## Conclusion

Patients with internal carotid artery aneurysms presenting with cranial neuropathy showed an 83% clinical improvement rate 12 months after flow diversion treatment. The rate increased over the first 12 months after treatment, but not thereafter; rather, it decreased slightly. Treatment within 6 months of symptom onset and aneurysmal regression were factors related to improvement. Transient worsening and worse symptoms 12 months after treatment were noted in 25% and 3% of patients, respectively. Aneurysm enlargement and the need for re-treatment may be associated with permanent worsening. Aneurysm size ≥20 mm was associated with transient worsening. Steroids may improve worsening symptoms.

10.1136/jnis-2022-019202.supp2 Supplementary data

## Data Availability

Data are available upon reasonable request. Any additional data regarding this submission can be requested from the corresponding author via email.
