# Supplementary material for: Onset-to-treatment time and aneurysmal regression predict improvement of cranial neuropathy after flow diversion treatment in patients with symptomatic internal carotid artery aneurysms
Source: J Neurointerv Surg. 2022 Jul 19;15(9):886–91. doi: 10.1136/jnis-2022-019202 (PMC10447392; doi:10.1136/jnis-2022-019202)
Supplement: Supplementary data [file jnis-2022-019202supp001.pdf]

BMJ Publishing Group Limited (BMJ) disclaims all liability and responsibility arising from any reliance placed on this supplemental material which has been supplied by the author(s)

Supplemental table 1 Symptom improvement by cranial nerve

	Improved	Not improved	P value
Cranial neuropathies overall	72	17	
Cranial nerve II	12 (17)	10 (59)	0.001
Cranial nerve III	23 (32)	4 (24)	0.571
Cranial nerve IV	0 (0)	1 (6)	0.191
Cranial nerve V	10 (14)	0 (0)	0.198
Cranial nerve VI	27 (38)	2 (12)	0.048

Supplemental table 2 Previous studies reporting cranial neuropathy after flow diversion treatment

References	No. of patients	Mean follow-up*	Improvement	Improvement of each cranial neuropathy		
				II	III + IV + VI	V
Tachi et al., 2020[26]	18	12	10/18 (56%)	3/5 (60%)	7/14 (50%)	
Wang et al., 2019[14]	22	25.5 (24-30)	12/22 (54%)	9/19 (47%)	5/7 (71%)	
Oishi et al., 2018[16]	40	13.5 (1-45)	18/40 (45%)	3/10 (30%)	15/28 (54%)	0/2 (0%)
Miyachi et al., 2017[8]	6	6	5/6 (83%)	NA	NA	
Silva et al., 2017[9]	64	NA	45/64 (71%)	45/64 (71%)		
Brown et al., 2016[11]	45	8.4 (0.1-21.5)	30/45 (67%)	6/10 (60%)	30/35 (86%)	
Sahlein et al., 2015[13]	39	6	25/39 (64%)	9/17 (53%)	17/27 (63%)	1/3 (33%)
Moon et al., 2014[12]	20	7 (4-28)	15/20 (75%)	2/3 (67%)	13/16 (81%)	3/4 (75%)
Tanweer et al., 2014[27]	19	NA	16/19 (84%)	NA	NA	
Zanaty et al., 2014[10]	51	14.5 (2.2-26.8)	47/51 (92%)	NA	NA	
Szikora et al., 2013[25]	16	NA (12-18)	15/16 (94%)	NA	NA	
O'Kelly et al., 2013[24]	27	NA	18/27 (67%)	5/9 (56%)	13/18 (72%)	
Current study	77	40 (12-72)	62/77 (81%)	12/22 (55%)	50/57 (88%)	10/10 (100%)
Total	444		318/444 (72%)	94/159 (59%)	150/202 (74%)	14/19 (74%)

*Expressed in months with range in parentheses. If range is not specified, all cases were evaluated at the time noted.

NA, not available

BMJ Publishing Group Limited (BMJ) disclaims all liability and responsibility arising from any reliance placed on this supplemental material which has been supplied by the author(s)

Supplemental Table 3 Univariate analysis for predictors of aneurysmal regression

Variables	+ regression	- regression	P value
No. of patients	33	33	
Age >65 yrs	20 (61)	18 (55)	0.804
Women	28 (85)	29 (88)	1.000
Hypertension	17 (52)	20 (61)	0.620
Dyslipidemia	14 (42)	11 (33)	0.612
Diabetes mellitus	1 (3)	3 (9)	0.613
History of smoking	14 (42)	7 (21)	0.112
Aneurysm size ≥20 mm	14 (42)	14 (42)	1.000
Aneurysm neck ≥8 mm	17 (52)	17 (52)	1.000
Steroid use	22 (67)	25 (76)	0.587
Adjunctive coiling	1 (3)	17 (52)	< 0.0001
Aneurysm occlusion*			
6 months	29 (88)	20 (61)	0.023
12 months	32 (97)	24 (73)	0.013

Values shown are medians with interquartile range or numbers with percentage.

*Aneurysm occlusion is defined as O'Kelly–Marotta grade C or D.

**Supplemental table 4** Relationship between volume embolization rate and resolution or improvement of cranial neuropathy in patients who underwent adjunctive coiling			
Variable	Improved	Unimproved	P value
No of Patients	9	9	
VER	11.6 (9.8-14.9)	17.3 (13.9-23.2)	0.0243
VER<13	6 (67)	1 (11)	0.0498

Values shown are medians with interquartile range or numbers with percentage.
